# Computing the noncentral-*F* distribution and the power of the *F*-test with guaranteed accuracy

**DOI:** 10.1007/s00180-016-0701-3

**Published:** 2016-12-08

**Authors:** Ali Baharev, Hermann Schichl, Endre Rév

**Affiliations:** 1 Faculty of Mathematics, University of Vienna, Oskar-Morgenstern-Platz 1, 1090 Vienna, Austria; 2 Budapest University of Technology and Economics, 1521 Budapest, Pf. 91, Budapest, Hungary

**Keywords:** Minimal detectable differences, ANOVA, Noncentrality parameter, Self-validating numerical method, Interval arithmetic

## Abstract

The computations involving the noncentral-*F* distribution are notoriously difficult to implement properly in floating-point arithmetic: Catastrophic loss of precision, floating-point underflow and overflow, drastically increasing computation time and program hang-ups, and instability due to numerical cancellation have all been reported. It is therefore recommended that existing statistical packages are cross-checked, and the present paper proposes a numerical algorithm precisely for this purpose. To the best of our knowledge, the proposed method is the first method that can compute the noncentrality parameter of the noncentral-*F* distribution *with guaranteed accuracy* over a wide parameter range that spans the range relevant for practical applications. Although the proposed method is limited to cases where the the degree of freedom of the denominator of the *F* test statistic is even, it does not affect its usefulness significantly: All of those algorithmic failures and inaccuracies that we can still reproduce today could have been prevented by simply cross-checking against the proposed method. Two numerical examples are presented where the intermediate computations went wrong silently, but the final result of the computations seemed nevertheless plausible, and eventually erroneous results were published. Cross-checking against the proposed method would have caught the numerical errors in both cases. The source code of the algorithm is available on GitHub, together with self-contained command-line executables. These executables can read the data to be cross-checked from plain text files, making it easy to cross-check any statistical software in an automated fashion.

## Introduction

The computational task that this paper deals with, when viewed from a high level of abstraction, is equivalent to solving univariate equations *with guaranteed accuracy*. Since roots of monotone functions are sought, the challenge is not in the root-finding, but in computing the function values. These intermediate computations are notoriously difficult to implement properly in floating-point arithmetic:

- Under- and overflow problems were reported by Benton and Krishnamoorthy (2003), Ding (1997), Helstrom and Ritcey (1985) independently of us;
- we also reveal in Baharev and Kemény 2008 that the algorithms of Norton (1983) and Lenth (1987) are exposed to over- and underflow issues, and that the Appendix 12 of Lorenzen and Anderson (1993, p. 374) is most likely bogus due to overflow;
- catastrophic round-off errors were reported by Frick (1990);
- drastically increasing computation time and hang-ups were observed by Chattamvelli (1995), Benton and Krishnamoorthy (2003);
- other noncentral distributions are similarly challenging, see for example Oliveira and Ferreira (2012).

If some of the intermediate computations suffer catastrophic loss of precision, the root-finding method can still succeed, and the final result presented to the user may nevertheless seem plausible. This was the case with the Appendix 12 of Lorenzen and Anderson (1993, p. 374): The errors in the intermediate computations remained unnoticed, despite the authors of the book being very diligent.

In theory, one could analyze the floating-point behavior of all the intermediate computations with pen and paper, and derive mathematically proven bounds on the final numerical error of the entire algorithm when implemented in floating-point arithmetic. However, as the above listed failures demonstrate, this task is too error-prone for humans to carry out for a non-trivial numerical algorithm. One way to mitigate this issue is to automate the numerical error analysis as much as possible: Interval arithmetic (to be discussed in Sect. 2) is a way to keep track of numerical errors automatically, and to guarantee that floating-point issues such as over- and underflow are noticed. However, interval arithmetic still requires that a human analyzes the floating-point behavior of all the basic operations and all the involved mathematical functions with pen and paper, and derives rigorous error bounds for all the possible floating-point inputs. It is still a huge win because “only” the smallest building blocks of the algorithm (basic operations and mathematical functions) have to be analyzed by a human, but not the entire algorithm as before: The numerical error analysis of algorithms built from the human-verified building blocks happens automatically because interval arithmetic keeps track of the floating-point errors as the algorithm is executed. In short, interval arithmetic reduces the amount of analysis that humans have to carry out with pen and paper, but it does not eliminate it. With interval arithmetic we essentially give the rest of the error analysis over to the computer, which then has to finish it as it executes the algorithm. It obviously has a runtime cost; in case of the proposed method, the runtime cost is negligible.

All of those algorithmic failures and inaccuracies that we can still reproduce today could have been prevented by simply cross-checking a sparse but sufficiently wide grid of values against the accurate values provided by the proposed method. By ‘sufficiently wide’ we mean that the grid of values spans the parameter range that is relevant for practical applications. In case of the noncentral beta distribution, we consider this range to be roughly the range spanned by Appendix 12 of Lorenzen and Anderson (1993, p. 374), i.e., shape parameters $a\in [0.5, 25]$ and $b\in [0.5, 500]$, type I and type II errors 0.05 and 0.10, respectively. If a numerical algorithm fails or is inaccurate, it typically happens not at isolated tiny regions of the parameter space but over a wide and continuous range, and especially at or near the extremes of the parameter ranges. Therefore, it is not necessary for the grid of cross-checked values to be dense; for cross-checking, it is sufficient if it has points near the extremes of the parameter ranges. We give numerical examples in Sect. 4 to support this claim.

The proposed method is limited to cases where the the degree of freedom of the denominator of the *F* test statistic is even. Although this is a limitation, it does not affect the usefulness of the proposed method significantly: All of those algorithmic failures and inaccuracies that we can still reproduce today could have been prevented by simply cross-checking against the proposed method.

To the best of our knowledge, the proposed method is the first method that can compute the noncentrality parameter of the noncentral-*F* distribution *with guaranteed accuracy* over a wide parameter range that spans the range relevant for practical applications. In our literature research we found the related self-validating numerical methods by Wang and Kennedy Wang and Kennedy (1990, 1992, 1994, 1995), out of which only Wang and Kennedy (1995) is directly relevant. These methods were published more than 20 years ago, and their source code is not publicly available. As we discuss in Baharev and Kemény (2008), the method in Wang and Kennedy (1995) is susceptible to over- and underflow: For example, it would over- or underflow due to the large value of the noncentrality parameter if we tried to compute the top right entry of Table 4 in contrast to the proposed method that has no difficulties there.

### Outline for the rest of the paper

The paper is structured as follows. The proposed method achieves guaranteed accuracy by applying interval arithmetic. In Sect. 2.1 we give an informal overview of interval arithmetic with an example, and the reader can compare and contrast it with ordinary floating-point arithmetic. Interval arithmetic is intentionally treated as a black box in Sect. 2.1: We only present what it delivers, but we do not discuss how. Then, in Sect. 2.2 we give a formal overview of how interval arithmetic guarantees rigorous error bounds. Sect. 3.1 derives the univariate equations that this paper is concerned with, Eqs. (9) and (11). Section 3.2 discusses how interval arithmetic and the interval Newton method can be applied to solve (9) and (11). The formal description of the proposed algorithm is presented in Sect. 3.3 with pseudo-code. We finally present in Sect. 4 two examples from the literature, where the intermediate computations involving the noncentral-*F* distribution went wrong silently, but the final result of the computations seemed nevertheless plausible, and erroneous results were published. Cross-checking against the proposed method would have caught the numerical errors in both cases.

## Interval arithmetic

### Automatic numerical error analysis with interval arithmetic: an example

The goal of this example is to demonstrate that interval arithmetic performs automatic numerical error analysis. The reader can think of interval arithmetic as a computationally cheap way to get guaranteed lower and upper bounds on the range of a function over a given domain of the variables. The obtained bounds are not necessarily sharp, but they are guaranteed to enclose the true range of the function despite the intermediate computations being carried out in floating-point arithmetic, and potentially suffer catastrophic loss of precision. Interval arithmetic can safely work with infinity, division by zero, etc., and automatically keeps track of the numerical error propagation throughout the intermediate floating-point computations.

We examine the numerical behavior of the following two functions:

1 $$\begin{aligned} f(n) = \left( 1 + \frac{1}{n}\right) ^n, \quad g(n) = \left( 1 - \frac{1}{n}\right) ^{-n}, \quad n \ge 2. \end{aligned}$$

It is known, see e.g. Li and Yeh (2013), that *f*(*n*) is monotone increasing, *g*(*n*) is monotone decreasing, ${\lim \nolimits _{n\rightarrow \infty }}f(n)= {\lim \nolimits _{n\rightarrow \infty }}g(n)=e$, and as a consequence $f(n)<e<g(n)$. If we carried out the computations in exact arithmetic, we would get tighter and tighter enclosures for *e* as *n* increases. However, we get the erratic results shown in Table 1 when the computations are carried out with 64 bit floating-point numbers on a computer. The source code of the example is available on GitHub at Baharev (2016) if the reader wishes to reproduce the numerical results, or analyze the implementation. For $k=9,10,11,12,15$, the supposed lower bounding *f*(*n*) values exceed the true value of *e*, where $n=10^k$; these are the rows with negative entries in the column for $e-f(n)$. For $k=9,12,14,15,17$, the supposed upper bounding *g*(*n*) values fall below the true value of *e*; these are the rows with negative entries in the column for $g(n)-e$. For $k=9,12,15$, the supposed lower bounding values exceed even the supposed upper bounding values, meaning that we did not even get an enclosure. The *f*(*n*) values are supposed to be increasing, but for $k=13,14,16,17$ it is clearly not the case. Similarly, *g*(*n*) is supposed to be monotone decreasing, but it is violated, e.g., at $k=13,16$.

**Table 1:** The numerical values of *f*(*n*) and *g*(*n*) defined in (1) when evaluated with 64 bit floating-point numbers on a computer where $n = 10^{k}$

*k*	*f*(*n*)	*g*(*n*)	$e-f(n)$	$g(n)-e$
1	2.59374246	2.86797199	0.12453937	0.14969016
2	2.70481383	2.73199903	0.01346800	0.01371720
3	2.71692393	2.71964222	0.00135790	0.00136039
4	2.71814593	2.71841776	0.00013590	0.00013593
5	2.71826824	2.71829542	0.00001359	0.00001359
6	2.71828047	2.71828319	0.00000136	0.00000136
7	2.71828169	2.71828196	0.00000013	0.00000013
8	2.71828180	2.71828186	0.00000003	0.00000003
9	2.71828205	2.71828175	−0.00000022	−0.00000008
10	2.71828205	2.71828205	−0.00000022	0.00000023
11	2.71828205	2.71828205	−0.00000022	0.00000022
12	2.71852350	2.71822170	−0.00024167	−0.00006013
13	2.71611003	2.71912720	0.00217179	0.00084537
14	2.71611003	2.71611003	0.00217179	−0.00217179
15	3.03503521	2.71611003	−0.31675338	−0.00217179
16	1.00000000	3.03503521	1.71828183	0.31675338
17	1.00000000	1.00000000	1.71828183	−1.71828183

See the text for discussion

Since the *f*(*n*) and *g*(*n*) functions are fairly simple, one could carry out a rigorous error analysis of these functions with pen and paper, and figure out the accuracy of the table entries. We will do this automatically with interval arithmetic.

Interval arithmetic takes the function $f(10^k)$, and the interval for *k* as input. (The interval for *k* is just the point interval [*k*, *k*] for each row in Table 1.) The output of the interval function evaluation is a rigorous enclosure of the range of $f(10^k)$ over [*k*, *k*]. In exact arithmetic, the range of $f(10^k)$ over the point interval [*k*, *k*] is obviously just a single real number, the value $f(10^k)$. However, we carry out the computations with 64 bit floating-point numbers on a computer. Interval arithmetic automatically keeps track of the numerical errors that occur during these computations, and we do not get a single real number but a pair of floating-point numbers as a result, that is, an interval enclosing the value of $f(10^k)$, the interval $[\underline{f}(10^k),\overline{f}(10^k)]$. These intervals are given in Table 2 for $k=1,2,\dots ,17$, together with the similarly computed $[\underline{g}(10^k),\overline{g}(10^k)]$.

**Table 2:** Rigorous enclosures of *f*(*n*) and *g*(*n*) defined in (1) where $n=10^k$

*k*	$\underline{f}(n)$	$\overline{f}(n)$	$\underline{g}(n)$	$\overline{g}(n)$	$e-\underline{f}(n)$	$\overline{g}(n)-e$
1	2.59374246	2.59374247	2.86797199	2.86797200	0.12453937	0.14969017
2	2.70481382	2.70481383	2.73199902	2.73199903	0.01346800	0.01371720
3	2.71692393	2.71692394	2.71964221	2.71964222	0.00135790	0.00136039
4	2.71814592	2.71814593	2.71841775	2.71841776	0.00013591	0.00013593
5	2.71826823	2.71826824	2.71829541	2.71829543	0.00001360	0.00001360
6	2.71828046	2.71828047	2.71828318	2.71828319	0.00000136	0.00000136
7	2.71828168	2.71828170	2.71828196	2.71828197	0.00000015	0.00000014
8	2.71828179	2.71828186	2.71828182	2.71828186	0.00000004	0.00000003
9	2.71828144	2.71828206	2.71828175	2.71828206	0.00000039	0.00000023
10	2.71827601	2.71828206	2.71827903	2.71828206	0.00000582	0.00000023
11	2.71822169	2.71828206	2.71825187	2.71828206	0.00006014	0.00000023
12	2.71791992	2.71852350	2.71822169	2.71852350	0.00036190	0.00024167
13	2.71611003	2.72214772	2.71611003	2.71912720	0.00217180	0.00084537
14	2.71611003	2.77709435	2.71611003	2.74643293	0.00217180	0.02815110
15	2.43069790	3.03503521	2.71611003	3.03503521	0.28758393	0.31675338
16	0.99999999	9.21143871	0.99999999	3.03503521	1.71828183	0.31675338
17	0.99999999	$4.399\times 10^9$	0.99999999	$6.632\times 10^4$	1.71828183	$6.632\times 10^4$

The enclosures were obtained with interval arithmetic, and $f(n)\in [\underline{f}(n),\overline{f}(n)]$ and $g(n)\in [\underline{g}(n),\overline{g}(n)]$ are guaranteed to hold

See the text for discussion

The wide intervals in Table 2, for example the rows for $k\ge 15$, are a clear sign of the intermediate computations suffering catastrophic loss of precision; it is guaranteed that with interval arithmetic we always get informed (through wide intervals) when this happens. We consider this one of the biggest advantages of this approach.

Despite the serious numerical difficulties for $k\ge 9$, the above discussed properties of *f*(*n*) and *g*(*n*) are still preserved in some form: (i) $\overline{f}(n)$ is monotone increasing, and $\underline{g}(n)$ is monotone decreasing, (ii) $\underline{f}(n)<e<\overline{g}(n)$ holds (unlike in Table 1, there are no negative entries in the last two columns of Table 2). The fact that these properties are preserved is not a coincidence either but the guaranteed behavior of interval arithmetic. However, note that we did not get tighter and tighter enclosures for *e* as *k* increased: The enclosure $[\underline{f}(10^k),\overline{g}(10^k)]$ reaches its minimum width at $k=8$, then the width starts increasing. We cannot blame interval arithmetic for this: Interval arithmetic is implemented on the top of 64 bit floating-point numbers, and unless one uses some extended precision arithmetic, *e* cannot be enclosed better with this simple approach. The tightest *verified* enclosure we got is [2.71828179, 2.71828186] for $k=8$; indeed, the correct value is $2.718281828\ldots$, and it is enclosed.

Let us emphasize again that for this simple example one could have derived bounds on the numerical errors of the entries in Table 1 with pen and paper. The advantage of interval arithmetic is that the numerical error analysis of the computations happens fully automatically, and therefore certain kinds of human errors are completely eliminated.

### A formal overview of interval arithmetic

*Interval arithmetic* is an extension of real arithmetic defined on the set of real intervals, rather than the set of real numbers. According to a survey paper by Kearfott (1996), a form of interval arithmetic perhaps first appeared in Burkill (1924). Modern interval arithmetic was originally invented independently in the late 1950s by several researchers; including Warmus (1956), Sunaga (1958) and finally Moore (1959), who set firm foundations for the field in his many publications, including the foundational book Moore (1966). Since then, interval arithmetic is being used to rigorously solve numerical problems.

Let $\mathbb {IR}$ be the set of all real intervals, and take $\mathbf {x}, \mathbf {y}\in \mathbb {IR}$. We set $\underline{x} := \inf {\mathbf {x}}$ and $\overline{x} := \sup {\mathbf {x}}$, such that $\mathbf {x}=[\underline{x},\overline{x}]$. Furthermore, the width of $\mathbf {x}$ is defined as ${\text {wid}}(\mathbf {x}) := \overline{x}-\underline{x}$, the radius of $\mathbf {x}$ as ${\text {rad}}(\mathbf {x}) := \tfrac{1}{2}{\text {wid}}(\mathbf {x})$, the magnitude of $\mathbf {x}$ as $|\mathbf {x}| := \max (\underline{x},\overline{x})$, and the mignitude of $\mathbf {x}$ as $\langle \mathbf {x}\rangle := \min \{|x|\mid x\in \mathbf {y}\}$. For $\mathbf {x}$ bounded we set the midpoint of $\mathbf {x}$ as $\check{x} := \tfrac{1}{2}(\underline{x}+\overline{x})$. We define the elementary operations for *interval arithmetic* by the rule $\mathbf {x}\diamond \mathbf {y}= \Box \{x \diamond y \mid x \in \mathbf {x}, y \in \mathbf {y}\}, \forall \diamond \in \{+, -, \times , \div , \hat{}~\}$, where $\Box S$ denotes the smallest interval containing the set *S*. (The symbol ‘$\Box$’ is a box, and it refers to the tightest interval hull, also called as box hull.) Thus, the ranges of the five elementary interval arithmetic operations are exactly the ranges of their real-valued counterparts. Although this rule characterizes these operations mathematically, the usefulness of interval arithmetic is due to the *operational definitions* based on interval bounds Hickey et al. (2001). For example, let $\mathbf {x}= [\underline{x}, \overline{x}]$ and $\mathbf {y}= [\underline{y}, \overline{y}]$, it can be easily proved that

$$\begin{aligned} \mathbf {x}+ \mathbf {y}= & {} [\underline{x}+\underline{y},\overline{x}+\overline{y}],\\ \mathbf {x}- \mathbf {y}= & {} [\underline{x}-\overline{y},\overline{x}-\underline{y}],\\ \mathbf {x}\times \mathbf {y}= & {} [\min \{\underline{x}\underline{y}, \underline{x}\overline{y}, \overline{x}\underline{y}, \overline{x}\overline{y} \}, \max \{\underline{x}\underline{y}, \underline{x}\overline{y}, \overline{x}\underline{y}, \overline{x}\overline{y} \}],\\ \mathbf {x}\div \mathbf {y}= & {} \mathbf {x}\times 1/\mathbf {y} if 0 \notin \mathbf {y}, where 1/\mathbf {y}= [1/\overline{y}, 1/\underline{y}],\\ \mathbf {x}^{\mathbf {y}}= & {} [\min \{\underline{x}^{\underline{y}}, \underline{x}^{\overline{y}}, \overline{x}^{\underline{y}}, \overline{x}^{\overline{y}} \}, \max \{\underline{x}^{\underline{y}}, \underline{x}^{\overline{y}}, \overline{x}^{\underline{y}}, \overline{x}^{\overline{y}} \}],~~\underline{y}>0, ~~\underline{x}\ge 0 . \end{aligned}$$

In addition, for a function $\varphi :\mathbb {R}\rightarrow \mathbb {R}$ and an interval $\mathbf {x}$ we define

$$\begin{aligned} \varphi (\mathbf {x}) := \Box \{\varphi (x)\mid x\in \mathbf {x}\}. \end{aligned}$$

Moreover, if a function *f* composed of these elementary arithmetic operations and elementary functions $\varphi \in \{\sin ,\cos ,\exp ,\log ,\ldots \}$, i.e., factorable function, is given, *bounds on the range* of *f* can be obtained by replacing the real arithmetic operations and the real functions by their corresponding interval arithmetic counterparts.

The finite nature of computers precludes an exact representation of the *reals*. In practice, the real set, $\mathbb {R}$, is approximated by a finite set $\bar{\mathbb {F}} = \mathbb {F}\cup \{-\infty , +\infty \}$, where $\mathbb {F}$ is the set of floating-point numbers. The set of real intervals is then approximated by the set $\mathbb {I}$ of intervals with bounds in $\bar{\mathbb {F}}$. The power of interval arithmetic lies in its implementation on computers. In particular, *outwardly rounded* interval arithmetic allows *rigorous enclosures* for the ranges of operations and functions. This makes a qualitative difference in scientific computations, since the results are now intervals in which the exact result is guaranteed to lie. Interval arithmetic can be carried out for virtually every expression that can be evaluated with floating-point arithmetic. However, two important points have to be considered: Interval arithmetic is only *subdistributive*, so expressions that are equivalent in real arithmetic differ in interval arithmetic, giving different amounts of overestimation (the amount by which the real range of the function over an interval and the result computed by interval arithmetic differ). Therefore, computations should be arranged so that overestimation of ranges is minimized. Readers are referred to Alefeld and Herzberger (1983), Neumaier (1990), Hickey et al. (2001), Jaulin et al. (2001) for more details on basic interval methods.

Let $f:\mathbf {x}\rightarrow \mathbb {R}$ be continuously differentiable, and assume the existence of $\hat{x}\in \mathbf {x}$ with $f(\hat{x}) = 0$, and let $\tilde{x}\in \mathbf {x}$. Then by the mean value theorem we get

$$\begin{aligned} f(\hat{x}) = 0 = f(\tilde{x}) + f'(\xi )(\hat{x}-\tilde{x}), \end{aligned}$$

for some $\xi \in \mathbf {x}$. Therefore,

$$\begin{aligned} \hat{x} = \tilde{x} - \frac{f(\tilde{x})}{f'(\xi )}. \end{aligned}$$

Now let $\mathfrak f'$ be an interval extension of $f'$ and $\mathfrak f$ be an interval extension of *f*, i.e. a specific expression for computing an enclosure for the range of *f* over a given input interval $\mathbf {x}$. Then by the properties of interval arithmetic we get

$$\begin{aligned} \hat{x} \in \tilde{x} - \frac{\mathfrak f(\tilde{x})}{\mathfrak f'(\mathbf {x})} =: N(\mathfrak f,\mathfrak f';\mathbf {x},\tilde{x}). \end{aligned}$$

The operator *N* is called univariate interval Newton operator. Using this operator we can define the *interval Newton iteration* as

2 $$\begin{aligned} \mathbf {x}^{(k+1)} = \mathbf {x}^{(k)}\cap N(\mathfrak f,\mathfrak f';\mathbf {x}^{(k)},\check{x}^{(k)}), \end{aligned}$$

starting with $\mathbf {x}^{(0)} = \mathbf {x}$. This iteration has the properties that whenever $\mathbf {x}^{(k)} = \emptyset$ for some *k*, then $\mathbf {x}$ does not contain a zero of *f*. Otherwise $\hat{x}\in \mathbf {x}^{(k)}$ for all *k*, and ${\text {wid}}(\mathbf {x}^{(k+1)}) = O({\text {wid}}(\mathbf {x}^{(k)})^2)$ locally under mild assumptions on *f* and $\mathbf {x}^{(0)}$. Furthermore, if for any *k* we find that $\mathbf {x}^{(k+1)} \subseteq \text {int}(\mathbf {x}^{(k)})$, i.e., that the interval Newton operator maps the box $\mathbf {x}^{(k)}$ into its interior, then $\mathbf {x}^{(k)}$ contains a unique zero of *f*.

The interval Newton operator requires an interval extension $\mathfrak {f}'$ of the derivative of *f*. For every factorable function *f* such an extension can be constructed using algorithmic differentiation techniques, e.g., see Berz et al. (1996), Griewank and Corliss (1991), Griewank and Walther (2008). For univariate functions the most efficient approach is via the algebra of differential numbers $\mathcal {D}_1:=\mathbb {R}\times \mathbb {R}$, equipped with the following basic operations: Let $df:=(f,f'), dg:=(g,g')\in \mathcal {D}_1$, and $\varphi :\mathbb {R}\rightarrow \mathbb {R}$. Define

3 $$\begin{aligned} \begin{aligned} df\pm dg&:= (f\pm g,f'\pm g'),\\ df\cdot dg&:= (f\cdot g, f'\cdot g + f\cdot g'),\\ df/dg&:= (f/g, (f'\cdot g - f\cdot g')/g^2),\quad g\ne 0,\\ df^{dg}&:= (f^g, f^g\cdot (g'\cdot \log (f)+g\cdot f'/f)),\quad f>0,\\ \varphi (df)&:= (\varphi (f),\varphi '(f)\cdot f'). \end{aligned} \end{aligned}$$

The set of real numbers is embedded in $\mathcal {D}_1$ by $r\mapsto (r,0)$.

If $\tilde{f}(x)$ is an expression representing *f* using arithmetic operations and elementary functions, we can use $\tilde{f}$ to calculate $(y,y') = \tilde{f}((x,1))$ on $\mathcal {D}_1$ by replacing the operations and elementary functions in $\tilde{f}$ by their counterparts on $\mathcal {D}_1$, and then $y' = f'(x)$.

This approach can be generalized to compute an interval extension $\mathfrak {f}'$ of $f'$ by defining the algebra of interval differential numbers $\mathbb {I}\mathcal {D}_1:=\mathbb {IR}\times \mathbb {IR}$ and introducing again the operations (3) on $\mathbb {I}\mathcal {D}_1$ now using interval arithmetic operations in the components of the interval differential numbers. Using this algebra and an expression $\tilde{f}$ for *f*, we get by computing $(\mathbf {y},\mathbf {y}') = \tilde{f}((\mathbf {x},1))$ an enclosure $\mathbf {y}'\supseteq f'(\mathbf {x})$ and thereby an interval extension $\mathfrak {f}'$ of $f'$.

## The proposed method

### Derivation of the formulas

The *F* test statistic used in the analysis of variance (ANOVA) problems follows the non-central *F* distribution when the null hypothesis is false, i.e., $F_0 = F_{nc}(\nu _1,\nu _2,\lambda )$, where $\nu _1$ and $\nu _2$ are the degrees of freedom of the nominator and the denominator, respectively, of the *F* test statistic; $\lambda$ is the non-centrality parameter. This non-centrality parameter is related to the size of effects. The probability of the Type II error $\beta$ (not detecting an effect) is

4 $$\begin{aligned} \beta = P\left[ F_0 \le F_{\alpha }(\nu _1,\nu _2)\right] = P\left[ F_{nc}(\nu _1,\nu _2,\lambda ) \le F_{\alpha }(\nu _1,\nu _2)\right] , \end{aligned}$$

where $\alpha$ is the significance level of the test. The power calculation consists of calculating the power ($Power=1-\beta$) for certain effect sizes. When it is used in the reversed way, the power is fixed (e.g. $Power=0.9$), and the size of the effect is calculated; this is considered as effect of detectable size, see Johnson and Leone (1977, p. 170) and  Lorenzen and Anderson (1993). Another application where the noncentral *F*-distribution arises is in coverages of Clopper-Pearson confidence intervals for binomial proportions with misspecified parameter *p* (Puza and O’Neill 2006a, b).

The cdf $F(w,\nu _1,\nu _2,\lambda )$ of the noncentral *F*-distribution with $\nu _1$, $\nu _2$ degrees of freedom and noncentrality parameter $\lambda$, and the cdf $I_x(a,b;\lambda )$ of the noncentral beta distribution with shape parameters *a* and *b* and noncentrality parameter $\lambda$ are related as follows:

5 $$\begin{aligned} F(w,\nu _1,\nu _2,\lambda )=I_x(a,b;\lambda ), \end{aligned}$$

where $a=\frac{\nu _1}{2}$, $b=\frac{\nu _2}{2}$, and $x=\frac{\nu _1w}{\nu _1w+\nu _2}$. We will work with the noncentral beta distribution from now on.

The incomplete noncentral beta function ratio $I_x(a,b;\lambda )$ for $0\le x\le 1, a>0, b>0, \lambda \ge 0$ is defined as

6 $$\begin{aligned} I_x(a,b;\lambda )= \sum _{i=0}^{\infty }\frac{e^{-(\lambda /2)}(\lambda /2)^i}{i!}I_x(a+i,b), \end{aligned}$$

where $I_x(a,b)$ is the usual incomplete beta function ratio,

7 $$\begin{aligned} I_x(a,b) =\frac{{\varGamma }(a+b)}{\varGamma (a)\varGamma (b)} \int \limits _{0}^{x} t^{a-1} (1-t)^{b-1}\mathrm {d}t , \end{aligned}$$

and $\varGamma (a)$ is the (complete) gamma function

8 $$\begin{aligned} \varGamma (a)=\int \limits _{0}^{\infty } t^{a-1} e^{-t}\mathrm {d}t,~~ a>0, \end{aligned}$$

see for example Johnson et al. (1995).

Equation (6) cannot be used directly for actual numerical computations. The proposed method uses the the closed formula

9 $$\begin{aligned} I_x(a,b)=x^a\left( 1+\sum _{n=1}^{b-1}\left( \prod _{m=1}^n \frac{a+m-1}{m}\right) (1-x)^n\right) \end{aligned}$$

by Singh and Relyea (1992) (misprint corrected by Chattamvelli (1995)) for the computation of the cdf of the central beta distribution, and the closed formula

10 $$\begin{aligned} I_x(a,b;\lambda )=e^{-(\lambda /2)(1-x)} \sum _{i=0}^{b-1}\frac{\left( (\lambda /2)(1-x)\right) ^i}{i!}I_x(a+i,b-i) \end{aligned}$$

by Sibuya (1967) (and later published by Johnson et al. (1995)) for the cdf of the noncentral beta distribution. The shape parameter *b* must be integer. It is very interesting that the evaluation of (10), using (9), is possible in finitely many steps, requiring only the four arithmetic operations, the power function, and the exponential function; the formulas do not depend on any function for computing statistical distributions, or other special functions.

Detectable differences for a specified type II error probability $\beta$ are determined by the noncentrality parameter $\lambda$ for which the cdf value of the noncentral beta distribution equals $\beta$

11 $$\begin{aligned} I_{x_{1-\alpha }}(a, b;\lambda ) = \beta , \end{aligned}$$

where $x_{1-\alpha }$ is the upper $\alpha$ quantile of the central beta distribution with shape parameters $a=\nu _1/2$ and $b=\nu _2/2$; $\alpha$ denotes the allowed type I error probability. When minimal detectable differences are sought, one solves (11) for $\lambda$, given $a,b,\alpha$ and $\beta$.

### Overview of the proposed method

To summarize Sect. 3.1, one can compute minimal detectable differences for a specified type II error probability $\beta$ in the *traditional* setting as follows. Given the shape parameters $a=\nu _1/2$ and $b=\nu _2/2$, and the allowed type I error probability $\alpha$, the upper $\alpha$ quantile $x_{1-\alpha }$ of the central beta distribution is computed first by solving $I_x(a,b)=1-\alpha$ for *x*, using (9) and the traditional Newton method. Then, (11) is solved for the noncentrality parameter $\lambda$ with the traditional Newton method, given $x_{1-\alpha }, a, b$, and $\beta$.

The proposed method follows these steps of the traditional setting but all the computations are carried out with interval arithmetic instead of ordinary floating-point arithmetic, and the interval Newton method is used instead of the traditional Newton method. As a consequence, there are notable differences. The traditional Newton method has two outcomes: It either reaches convergence or it does not. In contrast, the interval Newton method has three possible outcomes as we discussed in Sect. 2.2 formally; these outcomes are enumerated below informally as cases a, b, and c.

(a): All solutions are rigorously enclosed, and each enclosure contains a unique zero. The result is the list of these enclosures. (In our case, there can be at most one zero, i.e., the list has at most one element.)
(b): It is proved with mathematical rigor that the function cannot have any zeros in the initial interval.
(c): There is at least one enclosure among the resulting enclosures of zeros which may contain a zero but verification of existence and/or uniqueness of a zero in that particular enclosure failed.

The primary use case of the proposed method is cross-checking correctness of existing statistical software. The user first computes $x_{1-\alpha }$ and $\lambda$ with the statistical software to be checked, given $a=\nu _1/2$, $b=\nu _2/2$, $\alpha$, and $\beta$. The $x_{1-\alpha }$ and $\lambda$ values are floating-point numbers, or in other words, zero-width intervals. The initial intervals for the interval Newton method are then constructed by inflating these point intervals $x_{1-\alpha }$ and $\lambda$ such that they are centered around $x_{1-\alpha }$ and $\lambda$, respectively, but they have non-zero widths. (In this context, inflation refers to the width of the interval: The width of the point interval $x_{1-\alpha }$ is increased from zero to a strictly positive value.) If these initial intervals contain the true values, and the interval Newton method succeeds in proving it (case a), then the algorithm under test is at least as accurate as the radius of the initial intervals in that studied case. Analogously, if the initial intervals do not contain the theoretically correct value, and the interval Newton method reliably proves that (case b), then the accuracy of the algorithm under test is less than the radius of the initial intervals. The very rare but unfortunate case c, when reliable conclusion cannot be drawn and further investigation may be needed, can usually be remedied by simply changing (increasing) the radius of the initial intervals. We have not experienced case c when computing in the parameter range relevant for practical applications, e.g., when computing Tables 4 and 5; we only experienced case c when insane parameters were set.

The proposed method also works with, for example, [0, 1] as initial interval for $x_{1-\alpha }$. In other words, the proposed method works even in the complete absence of an approximate value for $x_{1-\alpha }$, however, this is not the anticipated use case.

### Formal description of the proposed algorithm

*Input.*: The input data of the proposed algorithm are $x_{1-\alpha }$ and $\lambda$, that the user wants to cross-check. In the anticipated use case, $x_{1-\alpha }$ and $\lambda$ come from an existing statistical software whose correctness is being checked.
*Step 1.*: The initial intervals $x_0$ and $\lambda _0$ are obtained from the inputs $x_{1-\alpha }$ and $\lambda$ by inflating them as follows:
  12 $$\begin{aligned}&x_0 = \left[ (1-\epsilon _x)x_{1-\alpha },~ (1+\epsilon _x)x_{1-\alpha }\right] , \text{ and } \lambda _0 = \left[ (1-\epsilon _{\lambda })\lambda ,~ (1+\epsilon _{\lambda })\lambda \right] ,\nonumber \\ \end{aligned}$$
  where the inflation parameters $\epsilon _x$ and $\epsilon _{\lambda }$ are sufficiently small user-defined real numbers, for example $10^{-6}$.
*Step 2.*: A narrow interval containing the theoretically correct value of $x_{1-\alpha }$ is computed with the interval Newton method, using (9). If the interval Newton method proves that $x_0$ is guaranteed *not* to have a solution, or the verification of a unique solution in $x_0$ fails, exit with the corresponding error message.
*Step 3.*: Equation (11) is solved for $\lambda$ with the interval Newton method, using (10) and (9). The possible outcomes are: A rigorous enclosure of the true value of $\lambda$ is obtained, or $\lambda _0$ is proved not to contain the correct value, or the verification of a unique solution in $\lambda _0$ fails. The algorithm finishes here.
*Output.*: The rigorous enclosures for $x_{1-\alpha }$ and $\lambda$ are printed if the interval Newton iteration is successful in both Step 2 and Step 3, or the corresponding error message if any of these steps fails.

We implemented this algorithm in C++, the source code is available on GitHub under Baharev (2016).

### Implementation details

As for the implementation details, the above algorithm is implemented in C++ using the C-XSC module nlfzero. C-XSC is available from http://www.xsc.de, and it is documented in the book of Hammer et al. (1995). C-XSC implements the interval Newton method in one variable using automatic differentiation. No higher precision internal data format is used. All computations are done using the IEEE double format (64 bit). Evaluation of (9) and (10) could be significantly speeded up since it involves several redundant operations. For example, it is possible to reduce the number of switches between rounding modes considering that $a>0$, $b>0$, and $0\le x\le 1$ always hold. However, (9) and (10) are used directly, and no efforts were made to decrease the computation time because we found it to be satisfactory.

The parameters are assumed to lie in the domain that is relevant for practical applications, roughly: $a \le 25, b \le 500$, and $0.01 \le \alpha , \beta \le 0.99$; the inflation parameters are also assumed to be sane, say $< 10^{-4}$. Violating these assumptions may cause performance degradation and the algorithm may start reporting verification failures, but incorrect results will never appear in the output.

## Numerical results

As we claimed in the introduction, all of those algorithmic failures and inaccuracies that we can still reproduce today could have been prevented by simply cross-checking against the proposed method; we now give two such examples. In both examples, the intermediate computations suffer significant loss of precision, but the final results presented to the user seem nevertheless plausible, making these kinds of numerical errors particularly harmful. A by-product of the first example is that the algorithm of Baharev and Kemény (2008), implemented on the top of the built-in functions of R, is proved to be accurate for 6 significant digits in the investigated cases with mathematical certainty.

The entire source code is available on GitHub at Baharev (2016). The computations have been carried out with the following hardware and software configuration. Processor: Intel(R) Core(TM) i5-3320M CPU at 2.60GHz; operating system: Ubuntu 14.04.3 LTS with 3.13.0-67-generic kernel; compiler: gcc 4.8.4, compiler optimization flag: -O3; C-XSC 2.5.4 configuration left on the default values given by the install script.

### Example 1: minimal detectable differences for general ANOVA models

Appendix 12 of Lorenzen and Anderson (1993, p. 374) tabulates the minimal detectable differences for general ANOVA models as a function of $\nu _1$ and $\nu _2$, with the type I and type II error probabilities fixed at $\alpha =0.05$ and $\beta =0.10$, respectively. All entries in Appendix 12 seem plausible; there is no obvious sign that some of the entries have no correct significant digits (for example that entry in Appendix 12 that corresponds to the top right entry of Table 4 of the present paper). The entries of Appendix 12 are most likely bogus due to floating-point overflow (Baharev and Kemény 2008).

The error could have been caught by simply cross-checking against the proposed method. The corrected form of Appendix 12 of Lorenzen and Anderson (1993, p. 374), i.e., Table 3 of Baharev and Kemény (2008), fully spans the parameter range that is relevant for practical applications. This table (except rows for which *b* is not integer, i.e., $b=0.5, 1.5, 2.5, 3.5$) is recomputed with the proposed method, and it is given as Table 4. The input values of $x_{0.95}$ and $\lambda$ are computed by the algorithm of Baharev and Kemény (2008), implemented in R R Development Core Team (2015) and available as the package fpow; the inflation parameter values $\epsilon _x$ and $\epsilon _{\lambda }$ are both set to $10^{-6}$.

Table 3 contains the solution of $I_x(a,b)=1-\alpha$ for *x*, given *a*, *b* and $\alpha =0.05$. Table 4 contains the solution of (11), where the verified $x_{0.95}$ is obtained from the previous step, that is, from Table 3. The overall computation required less than 9 seconds. The output of the proposed algorithm is $x_{0.95}$ verified up to 12 significant digits, and $\lambda$ verified up to 10 significant digits. (Only 6 digits are shown in the Tables 3 and 4.) The algorithm of Baharev and Kemény (2008), implemented on the top of the built-in functions of R, is also proved to be accurate for 6 significant digits in the investigated cases with mathematical certainty.

**Table 3:** The upper $\alpha = 0.05$ quantiles, the solution of $I_x(a,b)=1-\alpha$ for *x*

*b*	*a*								
	0.5	1	1.5	2	2.5	3	5	10	25
1	9.02500e-01	9.50000e-01	9.66383e-01	9.74679e-01	9.79692e-01	9.83048e-01	9.89794e-01	9.94884e-01	9.97950e-01
2	6.58372e-01	7.76393e-01	8.31750e-01	8.64650e-01	8.86622e-01	9.02389e-01	9.37150e-01	9.66681e-01	9.86158e-01
3	4.99474e-01	6.31597e-01	7.04013e-01	7.51395e-01	7.85230e-01	8.10745e-01	8.71244e-01	9.28130e-01	9.69022e-01
4	3.99294e-01	5.27129e-01	6.03932e-01	6.57408e-01	6.97399e-01	7.28662e-01	8.07097e-01	8.87334e-01	9.49692e-01
5	3.31756e-01	4.50720e-01	5.26623e-01	5.81803e-01	6.24472e-01	6.58739e-01	7.48632e-01	8.47282e-01	9.29506e-01
6	2.83463e-01	3.93038e-01	4.65976e-01	5.20703e-01	5.64102e-01	5.99689e-01	6.96463e-01	8.09135e-01	9.09126e-01
7	2.47316e-01	3.48164e-01	4.17435e-01	4.70679e-01	5.13741e-01	5.49642e-01	6.50188e-01	7.73308e-01	8.88911e-01
8	2.19284e-01	3.12344e-01	3.77834e-01	4.29136e-01	4.71285e-01	5.06901e-01	6.09138e-01	7.39886e-01	8.69067e-01
9	1.96926e-01	2.83129e-01	3.44972e-01	3.94163e-01	4.35104e-01	4.70087e-01	5.72619e-01	7.08799e-01	8.49712e-01
10	1.78687e-01	2.58866e-01	3.17294e-01	3.64359e-01	4.03954e-01	4.38105e-01	5.40005e-01	6.79913e-01	8.30912e-01
11	1.63528e-01	2.38404e-01	2.93680e-01	3.38681e-01	3.76883e-01	4.10099e-01	5.10752e-01	6.53069e-01	8.12701e-01
12	1.50733e-01	2.20922e-01	2.73308e-01	3.16340e-01	3.53157e-01	3.85390e-01	4.84396e-01	6.28099e-01	7.95094e-01
13	1.39791e-01	2.05817e-01	2.55557e-01	2.96734e-01	3.32202e-01	3.63442e-01	4.60549e-01	6.04844e-01	7.78091e-01
14	1.30326e-01	1.92636e-01	2.39958e-01	2.79396e-01	3.13568e-01	3.43825e-01	4.38883e-01	5.83155e-01	7.61683e-01
15	1.22059e-01	1.81036e-01	2.26143e-01	2.63957e-01	2.96893e-01	3.26193e-01	4.19120e-01	5.62893e-01	7.45857e-01
20	9.26567e-02	1.39108e-01	1.75534e-01	2.06725e-01	2.34411e-01	2.59467e-01	3.41807e-01	4.79012e-01	6.74797e-01
30	6.25175e-02	9.50339e-02	1.21191e-01	1.44090e-01	1.64826e-01	1.83943e-01	2.49305e-01	3.68153e-01	5.65062e-01
40	4.71693e-02	7.21575e-02	9.25215e-02	1.10553e-01	1.27053e-01	1.42414e-01	1.96078e-01	2.98634e-01	4.85211e-01
50	3.78708e-02	5.81551e-02	7.48160e-02	8.96715e-02	1.03353e-01	1.16167e-01	1.61545e-01	2.51097e-01	4.24830e-01
100	1.90711e-02	2.95130e-02	3.82269e-02	4.61073e-02	5.34614e-02	6.04365e-02	8.58514e-02	1.39660e-01	2.61259e-01
250	7.66110e-03	1.19114e-02	1.54926e-02	1.87595e-02	2.18331e-02	2.47712e-02	3.56731e-02	5.98536e-02	1.20972e-01
500	3.83600e-03	5.97355e-03	7.78040e-03	9.43349e-03	1.09931e-02	1.24879e-02	1.80690e-02	3.06519e-02	6.38108e-02

All given digits are verified with the last digit rounded to the nearest

**Table 4:** The noncentrality parameter $\lambda$, the solution to Eq. (11)

*b*	*a*								
	0.5	1	1.5	2	2.5	3	5	10	25
1	4.61803e+01	9.00517e+01	1.33936e+02	1.77823e+02	2.21712e+02	2.65601e+02	4.41161e+02	8.80065e+02	2.19678e+03
2	1.93236e+01	3.04220e+01	4.08997e+01	5.11554e+01	6.13048e+01	7.13948e+01	1.11490e+02	2.11206e+02	5.09746e+02
3	1.53086e+01	2.20966e+01	2.82383e+01	3.41350e+01	3.99085e+01	4.56104e+01	6.80824e+01	1.23556e+02	2.89087e+02
4	1.37822e+01	1.90179e+01	2.36054e+01	2.79378e+01	3.21380e+01	3.62590e+01	5.23587e+01	9.17573e+01	2.08805e+02
5	1.29870e+01	1.74388e+01	2.12434e+01	2.47874e+01	2.81929e+01	3.15140e+01	4.43773e+01	7.55652e+01	1.67745e+02
6	1.25009e+01	1.64830e+01	1.98196e+01	2.28917e+01	2.58210e+01	2.86621e+01	3.95760e+01	6.57902e+01	1.42832e+02
7	1.21736e+01	1.58437e+01	1.88700e+01	2.16290e+01	2.42420e+01	2.67637e+01	3.63771e+01	5.92546e+01	1.26087e+02
8	1.19383e+01	1.53865e+01	1.81924e+01	2.07289e+01	2.31168e+01	2.54110e+01	3.40954e+01	5.45774e+01	1.14042e+02
9	1.17611e+01	1.50436e+01	1.76849e+01	2.00552e+01	2.22750e+01	2.43990e+01	3.23869e+01	5.10640e+01	1.04947e+02
10	1.16228e+01	1.47769e+01	1.72907e+01	1.95324e+01	2.16217e+01	2.36137e+01	3.10600e+01	4.83276e+01	9.78285e+01
11	1.15120e+01	1.45636e+01	1.69759e+01	1.91149e+01	2.11002e+01	2.29868e+01	3.00000e+01	4.61356e+01	9.20991e+01
12	1.14212e+01	1.43892e+01	1.67186e+01	1.87739e+01	2.06743e+01	2.24748e+01	2.91336e+01	4.43398e+01	8.73842e+01
13	1.13454e+01	1.42440e+01	1.65045e+01	1.84902e+01	2.03200e+01	2.20489e+01	2.84125e+01	4.28416e+01	8.34334e+01
14	1.12812e+01	1.41211e+01	1.63236e+01	1.82505e+01	2.00207e+01	2.16891e+01	2.78028e+01	4.15723e+01	8.00727e+01
15	1.12262e+01	1.40158e+01	1.61686e+01	1.80453e+01	1.97645e+01	2.13810e+01	2.72807e+01	4.04832e+01	7.71776e+01
20	1.10375e+01	1.36562e+01	1.56397e+01	1.73453e+01	1.88905e+01	2.03304e+01	2.54976e+01	3.67467e+01	6.71490e+01
30	1.08550e+01	1.33096e+01	1.51311e+01	1.66726e+01	1.80510e+01	1.93210e+01	2.37807e+01	3.31188e+01	5.72176e+01
40	1.07660e+01	1.31411e+01	1.48842e+01	1.63462e+01	1.76437e+01	1.88312e+01	2.29461e+01	3.13414e+01	5.22527e+01
50	1.07132e+01	1.30415e+01	1.47383e+01	1.61535e+01	1.74032e+01	1.85419e+01	2.24526e+01	3.02856e+01	4.92627e+01
100	1.06093e+01	1.28456e+01	1.44516e+01	1.57748e+01	1.69308e+01	1.79737e+01	2.14819e+01	2.81960e+01	4.32297e+01
250	1.05479e+01	1.27301e+01	1.42828e+01	1.55519e+01	1.66527e+01	1.76392e+01	2.09095e+01	2.69549e+01	3.95540e+01
500	1.05276e+01	1.26919e+01	1.42270e+01	1.54783e+01	1.65608e+01	1.75288e+01	2.07203e+01	2.65432e+01	3.83154e+01

Probability of the type I and type II errors are 0.05 and 0.10 respectively

All given digits are verified with the last digit rounded to the nearest

### Example 2: comparing the accuracy of numerical algorithms

One of the goals of Table 1 of Chattamvelli and Shanmugam (1997) was to illustrate that their algorithm gives more accurate results than Frick’s algorithm (Frick 1990) for large values of $\lambda$. We reproduced selected rows of their table in Table 5, and extended it with an extra column showing the correct values up to 7 significant digits. This extra column was computed with the proposed method. The correct values (correct up to 7 significant digits) enabled us to give the number of correct significant digits in parentheses after each table entry. Table 5 proves, with mathematical rigor, that the algorithm of Chattamvelli and Shanmugam (1997) is actually slightly less accurate than Frick’s algorithm (Frick 1990) for those large values of $\lambda$ that are shown in Table 5. Since the precise details of the computations are not given in Chattamvelli and Shanmugam (1997), we cannot tell where the algorithm in Chattamvelli and Shanmugam (1997) suffers from a loss of precision.

**Table 5:** The cdf of the non-central beta random variable

*a*	*b*	$\lambda$	*x*	Frick (1990)	Chattamvelli and Shanmugam (1997)	Correct
5	5	54	0.8640	0.4563026 (7)	0.4563021 (5)	0.4563026
5	5	170	0.9560	0.6022421 (6)	0.6022353 (5)	0.6022422
10	10	54	0.8686	0.9187790 (6)	0.9187770 (5)	0.9187791
20	20	54	0.8787	0.9998677 (7)	0.9998655 (5)	0.9998677
20	20	250	0.9220	0.9641169 (5)	0.9641113 (4)	0.9641191

Except the last column, the values are taken from Table 1 of Chattamvelli and Shanmugam (1997); the last column with the correct values is computed with the proposed method. The number of correct significant digits is given in parentheses
